# Myocardial Salvage is Reduced in Primary PCI-Treated STEMI Patients with Microvascular Obstruction, Demonstrated by Early and Late CMR

**DOI:** 10.1371/journal.pone.0071780

**Published:** 2013-08-19

**Authors:** Shanmuganathan Limalanathan, Jan Eritsland, Geir Øystein Andersen, Nils-Einar Kløw, Michael Abdelnoor, Pavel Hoffmann

**Affiliations:** 1 Department of Cardiology Oslo University Hospital Ullevål, Oslo, Norway; 2 Department of Radiology, Oslo University Hospital Ullevål, Oslo, Norway; 3 Center for Clinical Research, Unit of Epidemiology and Biostatistics, Oslo University Hospital Ullevål, Oslo, Norway; 4 Center for Heart Failure Research, University of Oslo, Norway; 5 Center for Clinical Heart Research University of Oslo, Norway; Cliniche Humanitas Gavazzeni, Italy

## Abstract

**Objectives:**

This study evaluates the association between microvascular obstruction and myocardial salvage, determined by cardiac magnetic resonance performed both in the acute stage of myocardial infarction and after 4 months.

**Methods:**

In patients with acute ST-elevation myocardial infarction treated by primary percutaneous coronary intervention, myocardial salvage, infarct size, left ventricular volumes, and ejection fraction were assessed by early (1–4 days) and follow-up (4 months) cardiac magnetic resonance. These variables were related to the presence or absence of microvascular obstruction at early investigation. Myocardial salvage was determined by: (1) myocardium at risk and infarct size measured in the acute stage and (2) myocardium at risk, measured acutely, and infarct size measured after 4 months. Multivariate analyses were performed, adjusting for clinical confounders at baseline.

**Results:**

Microvascular obstruction was present in 49 of 94 included patients, (52%). Myocardial salvage was significantly reduced in patients with microvascular obstruction, compared to those without: 23% vs. 38%, measured acutely, and 39.8% vs. 65.4%, after 4 months (p<0.001). The presence of microvascular obstruction was significantly and independently associated with large infarct size, lower left ventricular ejection fraction, and larger left ventricular end-systolic volume.

**Conclusion:**

The presence of microvascular obstruction demonstrated by cardiac magnetic resonance early after infarction was associated with impaired myocardial salvage. This association was more marked when based on measurement of infarct size after 4 months compared to assessment in the acute stage.

## Introduction

Timely reperfusion by primary percutaneous coronary intervention (PCI) is the preferred treatment for patients suffering from acute ST-elevation myocardial infarction (STEMI) [1]. Reestablishment of flow in the infarct-related artery (IRA) is a prerequisite, but no guarantee, for normalization of myocardial microcirculation. Paradoxically, reperfusion of the IRA may result in death of potentially salvageable myocardium, contributing to the final infarct size by mechanisms collectively termed ischemia/reperfusion (I/R) injury [2], [3]. The pathophysiological mechanisms of I/R injury include intracellular perturbations and also, at the tissue level, luminal obliteration of microvessels by plugging of neutrophils, platelets, and artherothrombotic debris, as well as external compression by edema and haemorrhage, leading to microvascular obstruction (MVO) and “no reflow” or “low reflow” phenomena [4], [5].

In recent years, cardiac magnetic resonance (CMR) imaging has become an important tool in characterization of myocardial injury following an acute infarction. By different CMR modalities, variables like myocardial edema, infarct size, myocardial haemorrhage, and the presence of MVO, can be assessed early in the course of an acute myocardial infarction. From a clinical point of view, identification of reliable markers of long-term left ventricular (LV) function defined early in the course of STEMI could potentially have important implications regarding patient follow-up and treatment strategies. The demonstration of MVO by CMR has been shown to be associated with an adverse outcome regarding early and final infarct size, LV remodeling, and clinical long-term prognosis [6]–[14]. Based on data from CMR performed 1–4 days after STEMI, Eitel and co-workers reported an association between impaired myocardial salvage and the presence of MVO [15]. However, after reperfusion infarct size shrinks over time [12], [16], [17]. Lønborg and co-workers assessed myocardial salvage based on myocardium at risk measured in the acute stage and infarct size determined after 3 months in a STEMI population [18]. Thus, myocardial salvage calculated from myocardium at risk and final infarct size measured by CMR during the acute and late stages of STEMI, respectively, may be a more relevant reflection of acute STEMI treatment than salvage determined by a single, early CMR.

In the present study we determined myocardial salvage in 2 ways, based on CMR performed in the acute stage of STEMI as well as after 4 months. First, calculation of salvage was based on myocardium at risk and infarct size measured in the acute stage and, second, salvage was calculated from myocardium at risk, measured acutely, and final infarct size, measured at 4 months. Our aims were to compare myocardial salvage at the two time points and relate the results to the presence or absence of MVO at early CMR.

## Materials and Methods

The study population consisted of patients with STEMI treated by primary PCI within 6 hours, consecutively recruited from the ongoing POSTEMI study [19]. The POSTEMI study protocol has been approved by the Regional Committee for Medical Research Ethics, South- East, Norway, Ref. No. S-08421d, 2008/10614, and the trial is registered at ClinicalTrials.gov, NCT. No. PO 1506. All patients in the POSTEMI study gave written consent to store personal information in the hospital database for research purposes.

Patients with previous myocardial infarction, renal failure, collateral flow to the ischemic myocardium, unstable patients in cardiogenic shock and patients with pulmonary congestion or severe hypotension were excluded. An occluded IRA [Thrombolysis in Myocardial Infarction (TIMI) flow 0 or 1] with successful recanalization (TIMI flow 2 or 3) after first balloon inflation had to be demonstrated. All patients received aspirin 300 mg, clopidogrel 600 mg, and heparin 70 IU/kg (maximum 7000 IU) before or during PCI and the glycoprotein IIb/IIIa inhibitor eptifibatide was given during and after angioplasty, using a standard regimen of weight-adjusted bolus followed by infusion for minimum 12 hours [20]. Further treatment was given according to current guidelines for management of STEMI [1]. Blood samples were drawn for analysis of troponin T and CK-MB, and repeated until peak values. Two CMR examinations were performed, one early, 1–4 days after STEMI, and one at 4 months follow-up.

### CMR Protocol

CMR was performed on a 1.5 T scanner (Philips Intera, release 11, Best, the Netherlands), using five element synergy-cardiac coil and vector-based ECG. LV was scanned in two and four chamber long axis view using balanced fast field echo sequences for functional analysis, and short axis images were acquired for complete LV volume analysis. The scanning parameters were set to slice thickness 8 mm, slice gap 0 mm, and 25 heart phases.

T2 weighted imaging was performed in cardiac short axis plane using black blood inversion recovery fast spin echo sequences. To improve signal-to-noise ratio, a slice thickness of 15 mm was chosen and in total 5 slices were scanned from the apex to the basis of LV [21].

The late gadolinium enhancement (LGE) study was performed 15 minutes after contrast injection in two and four chamber long axis view and in short axis view, using 3D T1 enhanced gradient echo technique with inversion prepulses, covering the whole LV. A dose of 0.15 mmol/kg, gadolinium-DTPA 469 mg/ml (Magnevist, Schering AG, Germany) was injected using Spectris power injector. Slice thickness was 6 mm and slice gap was set to 0. After performing a “Look-Locker sequence”, optimal inversion time was chosen to suppress signal of normal myocardium.

### CMR Analysis

Image analysis was performed on View Forum workstation (Philips Medical Systems). Left ventricular ejection fraction (LV EF) was calculated by assessment of the volumes of the endocardial contours in diastole and systole of the short axis images. The included slice closest to the mitral valve plane had myocardium in at least 2/3 of the LV circumference. To obtain the volume of LV myocardium, epicardial tracing was done in diastole.

T2 weighted images were used to quantify the myocardium at risk. Myocardium with a signal intensity (SI) of more than 2 standard deviations above the SI in remote non-infarcted myocardium was considered as the “myocardium at risk”. The area indicated was also manually traced and compared to the area of the whole short axis slice.

On LGE images the size of the infarction was assessed, drawing the contour of the infarcted area in all short axis slices. The volume of the infarction was related to the total LV myocardial volume. Further, short axis slices corresponding to the slices used in the T2 study were chosen and the hyperintense area (>2 SD above SI in remote, normal myocardium) in these slices was traced. This hyperintense, infarcted area was then related to the myocardium at risk seen at T2 imaging.

**Figure 1:**
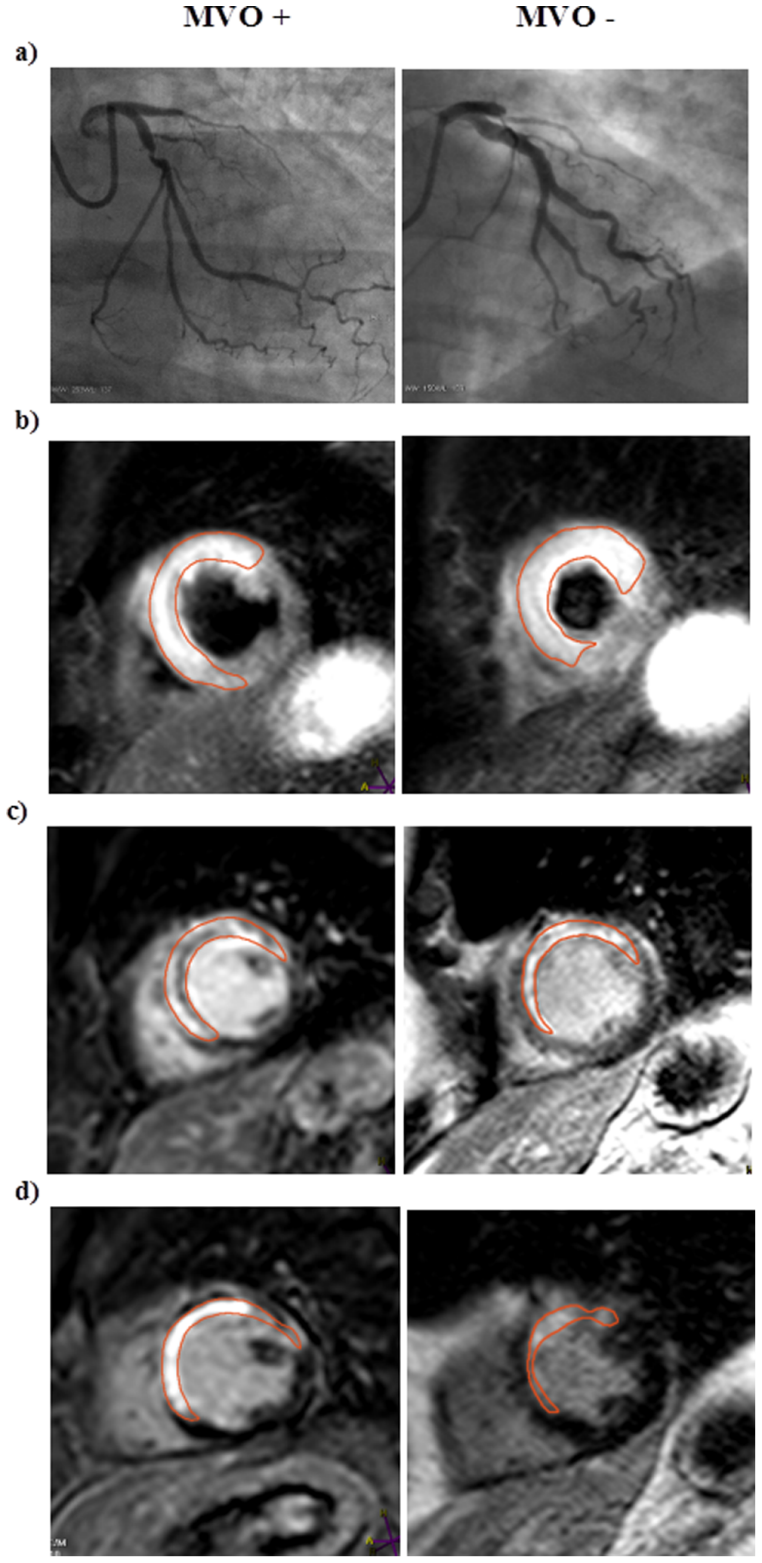
Coronary angiography and CMR images in the presence or absence of microvascular obstruction. **a)** Coronary angiography images showing proximal occlusion of the left anterior descending artery, **b)** CMR demonstrating short axis T2 weighted imaging of myocardium at risk (early CMR), **c)** short axis late enhancement imaging (early CMR), and **d)** short axis late enhancement imaging (late CMR), in patients with (MVO +) or without (MVO -) microvascular obstruction. Early CMR was performed 1–4 days after primary PCI and late CMR was performed 4 months later.

MVO was defined as a dark area in the centre of the hyperintense area in infarcted myocardium, assessed in late enhancement images. On manual tracing of infarcted area, the MVO dark area was included. Myocardial salvage (%) was defined as [(myocardium at risk –infarct size): myocardium at risk] x 100 [15]–[18], [22], [23]. Salvage was determined based on myocardium at risk and infarct size measured in the acute stage and, secondly, based on myocardium at risk (from the acute CMR) and infarct size after 4 months. Representative images showing coronary angiography, myocardium at risk, and final infarct size (by early and follow-up CMR) in patients with and without MVO are shown in Figure 1.

### Epidemiological Design and Statistics

Continuous variables are presented as median values with interquartile range. Categorical variables are presented as proportions. Differences between groups were analyzed by Mann-Whitney test for continuous variables and chi-square test for categorical variables in univariate analyses.

The study design was a prospective cohort with exposition, the presence of MVO or not, and different endpoints determined by early and late CMR (large infarct size, LV EF, LV volumes, changes in LV EF and LV volumes, and myocardial salvage).

For the endpoint “large infarct size”, defined as relative infarct volume ≥25.4% of LV myocardial volume (≥75 percentile) at 4 months, we estimated the crude odds ratio (OR) for the association between presence of MVO and this endpoint. Stratification analysis using the Mantel-Haenszel method was used to highlight potential effect modification by Breslow-Day test of heterogeneity and to quantify potential confounders. A multivariate logistic regression analysis on the association between MVO and the risk of developing a large final infarct size, including a backward elimination procedure, was performed to adjust for multiconfounders [24]. Because peak troponin value is known to be strongly correlated with infarct size, this variable was not included in the model.

The association between the presence or absence of MVO and the changes in EF and in LV volumes was analyzed by the covariance model (ANCOVA), controlling for baseline variables and confounders [25].

Analysis of a possible association between the presence of MVO and myocardial salvage was performed by a multivariate linear regression model, controlling for potential confounders [25]. The EpiInfo (version 3.5.1, Centers for Disease Control and Prevention, Atlanta, GA) and SPSS (version 15.0, SPSS Inc, Chicago, Ill) softwares were used throughout. A p-value <0.05 was considered statistically significant.

## Results

**Figure 2:**
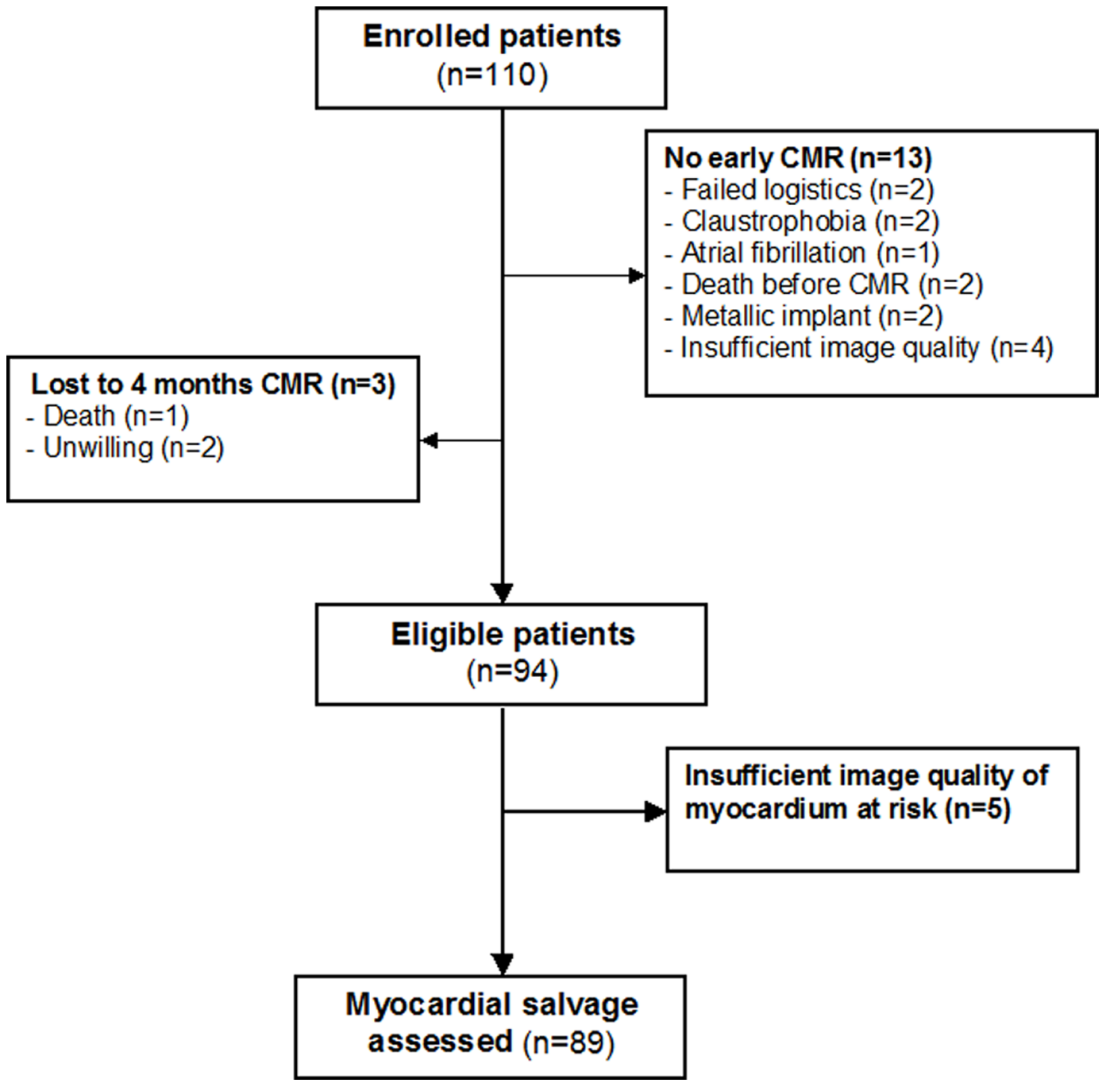
Study flow - diagram of included patients.

**Figure 3:**
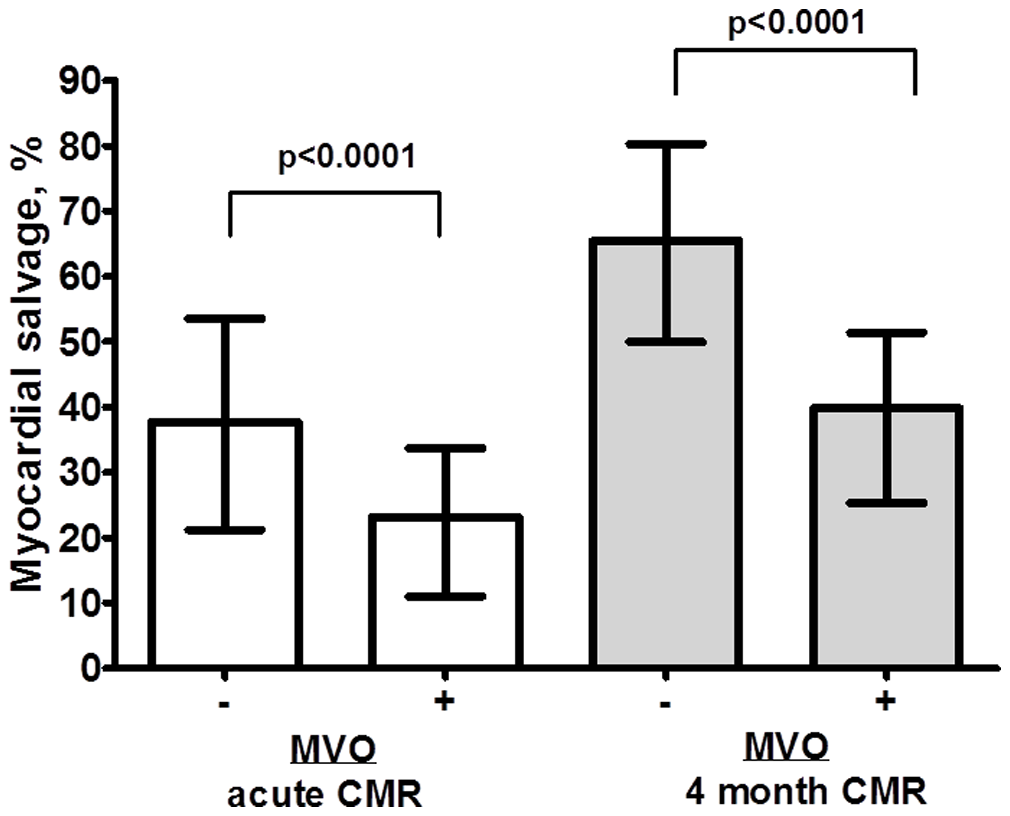
Myocardial salvage in patients with or without microvascular obstruction (MVO). Myocardial salvage, in patients with (MVO +) or without (MVO−) microvascular obstruction, assessed both by early and late CMR, respectively (percent, median values, and interquartile range).

A flow-chart of consecutive STEMI patients included in the present study is shown in Figure 2. A total of 94 patients, median age 62 (range 52–68) years, 84% males, were included in the analyses. Myocardial salvage was not calculated in 5 patients due to insufficient image quality. Baseline characteristics of patients with and without MVO are summarized in Table 1. The first CMR examination was performed at a median of 2.2 (range 1–4) days after revascularization. MVO was demonstrated in 49 patients (52%). In patients with MVO, a larger part of LV myocardium was at risk than in patients without MVO. Also, LV EF was lower and LV volumes were higher in the presence of MVO (Table 2), all p-values <0.001. Myocardial salvage, assessed from early CMR as well as after 4 months, was significantly lower in patients with MVO compared to those without (Table 2, Figure 3). During 4 months follow-up, infarct size decreased in both groups, but to greater extent in the group without MVO, leading to a more marked group difference in myocardial salvage. When analyzing the association between the presence or absence of MVO and the endpoint myocardial salvage, determined after 4 months, by a multivariate linear regression model, adjusting for door-to-balloon time, anterior wall infarction, and gender, 38% of the variance (R^2^ = 0.38) was attributable to a linear relationship between myocardial salvage and MVO. The association between MVO and myocardial salvage was highly significant also after adjustment for covariates [β/SE (β)] = −6.93, p<0.001.

**Table 1:** Patient baseline characteristics according to absence (MVO−) or presence (MVO +) of microvascular obstruction (MVO). Data are presented as median values (interquartile range) or numbers (percentage).

	MVO – (n = 45)	MVO+(n = 49)	p - value
Age (years)	61 (53–69)	62 (53–68)	0.84
Male gender	34 (76)	45 (92)	0.03
Treated hypertension	14 (31)	14 (29)	0.78
Diabetes	3 (7)	2 (4)	0.46
Current smoking	22 (49)	22 (45)	0.43
Symptom-to-balloon time (minutes)	171 (113–248)	170 (126–248)	0.71
Door-to-balloon time (minutes)	36 (29–45)	32 (28–39)	0.09
Systolic blood pressure* (mmHg)	145 (130–167)	140 (130–165)	0.88
Heart rate*(beats/minute)	76 (62–85)	78 (72–86)	0.28
LAD infarction	20 (45)	33 (68)	0.06
LCX infarction	5 (11)	5 (10)	
RCA infarction	20 (44)	11 (22)	
Pre-PCI TIMI flow 0	42 (93)	47 (96)	0.46
Pre-PCI TIMI flow 1	3 (7)	2 (4)	
Post- PCI TIMI flow 2	0	3 (6)	0.14
Post- PCI TIMI flow 3	45 (100)	46 (94)	
Bare metal stent	39 (89)	44 (90)	0.85
Drug eluting stent	5 (11)	5 (10)	
Average time to early CMR (days)	2.3 (2–3)	2.3 (1–2)	0.60
Peak troponin T (ng/L)	3431 (2283–5826)	8629 (5485–12988)	<0.0001

* at admission.

**Table 2:** Myocardium at risk, infarct size, left ventricular ejection fraction and volumes, and myocardial salvage in patients with and without microvascular obstruction (MVO). Data are presented as median values (interquartile range).

	Early CMR		Late CMR	
	MVO−	MVO+	MVO−	MVO+
Myocardium at risk (% of left ventricle)	37.1 (29.8–46.9)	47.7 (43.6–55.3)		
Infarct size (% of left ventricle)	11.6 (6.5–15.6)	24.0 (17.0–31.9)	6.6 (2.8–12.5)	17.7 (14.2–25.6)
Ejection fraction (%)	55 (50–62)	47 (41–53)	64 (59–70)	50 (43–54)
LV EDV^a^ (ml)	138 (118–167)	175 (156–198)	141 (124–176)	191 (158–219)
LV ESV^b^ (ml)	58 (47–78)	92 (72–114)	59 (43–68)	97 (72–122)
Myocardial salvage^c^ (%)	38 (21.2–53.6)	23 (11.6–33.7)	65.4 (50.5–79.6)	39.8 (25.3–51.4)

a LV EDV: Left ventricular end-diastolic volume.

b LV ESV: Left ventricular end-systolic volume.

c Myocardial salvage = [(Myocardium at risk – Infarct size)/Myocardium at risk]×100.

Measured early as well as after 4 months, LV volumes were significantly larger, whereas LV EF was lower in patients with, compared to patients without MVO, respectively (Table 2). We also analyzed the changes in these variables between baseline and 4 months. In univariate analyses, patients with MVO had less improvement in EF than patients without MVO. The change in LV end-diastolic volume (LV EDV) showed no significant group difference, but the change in LV end-systolic volume (LV ESV) was significantly different in the two groups.

The crude OR for developing a large myocardial infarction, measured after 4 months, in patients with MVO compared to patients without MVO at early CMR was 17.5 (95% CI, 3.8–80.5, p = 0.001). In order to adjust for covariates influencing the association between MVO and risk of large infarct size, we performed a multivariate analysis. No effect modification was found. The following variables were identified as potential confounders: anterior wall infarction, gender, door-to-balloon time, and myocardium at risk, and all except gender were included in the final model. The adjusted OR for developing a large infarction in the presence of MVO was 14 (95% CI, 2.3–93.5, p<0.001). A strong association between MVO and LV ESV as well as LV EF was shown, ([β/SE (β)] = 3.20, p<0.0001 and [β/SE (β)] = −5.48, p<0.0001) respectively, when adjusted in the ANCOVA model for the possible confounding effects of door-to-balloon time, anterior infarction, myocardium at risk, gender, and baseline LV ESV. However, there was no significant association between the presence of MVO and 4 months LV EDV after adjustment for the same covariates [β/SE (β)] = 1.04, p = 0.299.

During 4 months follow-up, none of the patients had re-infarction or stroke. Ten patients were re-hospitalized for congestive heart failure or unstable angina, 7 in the group without MVO and 3 in the group with MVO (p = 0.13).

## Discussion

In the present study, we found a strong, independent, and robust association between MVO and impaired myocardial salvage. Previously, an association between impaired myocardial salvage and MVO in STEMI patients has been reported, based on a single, early CMR [15]. As infarct size diminishes during the first weeks to months after STEMI [12], [16], [17], assessment of myocardial salvage based on myocardium at risk measured in the acute stage and infarct size measured in the late stage of STEMI might be a more relevant reflection of the STEMI treatment [18]. Moreover, the increase in LV EF from baseline to 4 months was less pronounced in patients with MVO, as compared to those without. Thus, the presence of MVO, as demonstrated by a CMR study during the first few days after STEMI, is a marker of an unfavorable recovery over time of jeopardized myocardium. MVO was also associated with a large final infarct size, and this association was still highly significant after adjustment for other markers of outcome, including myocardium at risk. Of note, none of our patients had collateral flow to IRA and, except for 3 patients with TIMI 2-flow, all patients in our study had normalization of macrovascular flow (TIMI 3) after PCI.

Our results confirm and extend previous reports on the association between MVO and variables of LV function after PCI-treated STEMI [9]–[12], [15]. In patients with presence of MVO, we also found larger LV ESV and change in LV ESV between baseline and 4 months than in patients without MVO. However, in contrast to other reports [7]–[9], [12], [13], after adjustment for confounders, the change in LV EDV was not significantly different in the 2 groups. Other baseline variables, including myocardium at risk, were of importance for the remodeling process, as expressed by a significant association with LVEDV. This observation corresponds with the results of Lund and co-workers [11], concluding that infarct size measured by early CMR is a stronger predictor of remodeling than MVO.

Some investigators have calculated the size and extent of MVO and related this to LV outcome variables [9], [14]. However, according to Nijveldt and co-workers [9], it is the presence and not the extent of MVO, which is the more important marker for adverse LV function. Consequently, we dichotomized the patients with respect to presence or absence of MVO.

By CMR the presence of MVO can be assessed by first pass perfusion from early gadolinium enhancement or from the LGE imaging phase (“persistent MVO”) [6], [10], [14], [20], [26]. These imaging modalities probably reflect different pathophysiological processes [9], [26] First pass perfusion imaging or early gadolinium enhancement is considered the more sensitive way to diagnose MVO by CMR [9], [10], [26], whereas persistent MVO probably is seen as an expression of a more severely injured microvasculature [26]. As persistent MVO has been reported to correlate with LV functional outcome and also with later clinical events [9], [14], we selected this variable in the present study. The frequency of MVO among STEMI patients will depend on time to reperfusion, and thus infarct size, as well as timing of imaging, and the modality used to define MVO [12]. We found an incidence of MVO of 52% in our patients, which is in the same range as other investigators have described [9]–[11], [14].

Myocardial salvage assessed by CMR has been identified as a predictor of clinical outcome [15], [22]. Moreover, the presence of MVO is a predictor of later cardiovascular events, even after adjusting for other determinants, including infarct size [6], [14]. Very few of our patients had clinical events during the 4 months follow-up, reflecting that unstable patients were not included in the present study. Therefore, we could not relate the presence of MVO or other baseline variables to the clinical outcome during this time period. However, as proposed by others, variables derived from CMR, like infarct size and myocardial salvage, can be regarded as relevant surrogate endpoints in moderate-size clinical trials on STEMI treatment [17].

### Limitations

The patients included in this study were recruited from an ongoing trial, investigating the effect of postconditioning during primary PCI on final infarct size. Thus, they were selected according to the inclusion and exclusion criteria of the trial [19]. We do not know the possible impact of postconditioning on MVO. In the present study we have demonstrated an association between the presence of MVO and myocardial salvage, LV EF, and infarct size, independent of the pathophysiological mechanisms behind MVO. A putative influence of postconditioning on these mechanisms cannot be excluded, inferring that postconditioning may be a hidden confounder. The patients sample included in the present study was relatively small and few clinical events were recorded during follow-up. Consequently, we could not relate occurrence of MVO to clinical events.

In summary, in PCI-treated STEMI patients we have demonstrated that the presence of MVO was associated with reduced myocardial salvage in the acute stage and the reduction was more marked when based on measurement of infarct size after 4 months. Thus, a CMR study early in the course of STEMI may yield information useful for the treatment and follow-up strategies of such patients.
